# Mesenchymal Stem Cell-Based Therapies Applied in Neurological Diseases: A Systematic Review

**DOI:** 10.3390/biomedicines14020475

**Published:** 2026-02-21

**Authors:** Ana Trabulo, Patrícia Sousa, Rui Alvites, Ana Colette Maurício

**Affiliations:** 1 Departamento de Clínicas Veterinárias, Instituto de Ciências Biomédicas de Abel Salazar (ICBAS), Universidade do Porto (UP), Rua de Jorge Viterbo Ferreira, No. 228, 4050-313 Porto, Portugal; anatrabulop@gmail.com (A.T.); pfrfs_10@hotmail.com (P.S.); ruialvites@hotmail.com (R.A.); 2 Rede de Química e Tecnologia (REQUIMTE), Instituto de Ciências, Tecnologias e Agroambiente da Universidade do Porto (ICETA), Rua D. Manuel II, Apartado 55142, 4051-401 Porto, Portugal; 3 Associate Laboratory for Animal and Veterinary Science (AL4AnimalS), 1300-477 Lisboa, Portugal; 4 Faculdade de Engenharia, Universidade do Porto, Rua Dr. Roberto Frias, 4200-465 Porto, Portugal; 5 Instituto Universitário de Ciências da Saúde (IUCS), Cooperativa de Ensino Superior Politécnico e Universitário (CESPU), Avenida Central de Gandra 1317, 4585-116 Paredes, Portugal

**Keywords:** mesenchymal stem cells, secretome, extracellular vesicles, neurodegenerative diseases, regenerative medicine

## Abstract

**Background/Objectives**: Neurodegenerative diseases (NDs) have a severe impact on patients’ quality of life, and effective treatments remain limited. As the focus is on treating the symptoms, the root cause of the problem is commonly not addressed. Mesenchymal stem cells show an emerging potential due to the ability for self-renewal combined with their capability for differentiation into various cell lines, which makes them a strong candidate for regenerative therapies in general, and for application in neurological issues in particular. This article provides an overview of the safety, efficacy, and challenges associated with the use of mesenchymal stem cells (MSCs) and their derived secretome in clinical and preclinical models of Alzheimer’s disease (AD), Parkinson’s disease (PD), Huntington’s disease (HD) and amyotrophic lateral sclerosis (ALS). **Methods:** A systematic search was conducted on PubMed to identify published studies providing clinical and preclinical evidence on the use of MSCs in neurodegenerative disorders. **Results**: Overall, the literature consistently indicates that MSCs and their derivatives exert disease-modifying effects across multiple NDs. Across AD, PD, HD and ALS, preclinical studies uniformly report improvements in behavioural outcomes, attenuation of neuroinflammation, and neuroprotective effects, largely mediated by MSCs’ paracrine signalling rather than direct cell replacement. Clinical studies to date consistently support the safety and feasibility of MSC-based therapies, while efficacy signals remain modest, heterogeneous and predominantly short-term, highlighting the need for larger, well-controlled trials. **Conclusions**: Integration of genetic engineering, preconditioning, and EV technology may represent an emerging therapeutic approach that may complement existing neuroregeneration treatments, offering a scalable and minimally invasive frontier to improve long-term clinical outcomes in patients with AD, PD, HD, and ALS.

## 1. Introduction

Neurodegenerative diseases such as Alzheimer’s disease (AD), Parkinson’s disease (PD), Huntington’s disease (HD) and Amyotrophic Lateral Sclerosis (ALS) represent a significant and growing burden on global healthcare systems, affecting millions of individuals by imposing substantial challenges to their quality of life, functional independence, and overall mortality rates [1,2,3]. This range of diseases spans an acute-to-chronic spectrum and affects, in particular, the central nervous system (CNS)—the brain (cerebrum, cerebellum and brainstem), retina, and spinal cord, the main organs responsible for processing all the primary body’s functions [4,5]. This system is composed of two major cellular compartments: neurons and glial cells. Neurons are electrically active cells, responsible for the generation and propagation of action potentials [6], creating a highly synchronised synaptic network that ensures cognitive, motor and sensory functions. Their function is supported by glial cells—astrocytes, microglia, oligodendrocytes and NG2 glia—which provide metabolic balance, immune surveillance and myelination to ensure rapid conduction of nervous impulses [7]. All these cells are fully interconnected in functional and physiological terms, and therefore any change in one of these cells may affect others [8].

Neurodegenerative diseases share common pathophysiological mechanisms that result in progressive and irreversible neuronal dysfunction in different CNS regions. While the abnormal aggregation and accumulation of misfolded proteins represent a central hallmark for NDs, additional processes can be involved in their pathogenesis. The specific clinical phenotype derives from the type of protein involved and the particular brain regions where the protein aggregation predominates [9]. The accumulation of misfolded protein results in neuronal homeostasis disruption, associated with mitochondrial dysfunction, increased production of reactive oxidative species (ROS) and oxidative stress, ultimately damaging key cellular components such as lipids, proteins and DNA [10]. Consequently, the gradual deterioration of neurons and glial cells in these tissues leads to a loss of specific neurological functions, such as memory, movement and cognition [4]. Beyond the cerebral consequences, neurodegeneration can also affect other regions of the CNS, producing characteristic functional deficits: in the cerebellum, neurodegeneration is primarily associated with motor dysfunction as well as cognitive and affective disturbances. Even though cerebellar involvement is not considered the main pathological feature in AD and PD, cerebellar atrophy [11,12] has been linked to motor and cognitive impairment, including deficits in learning, working memory and emotional dysregulation [13,14]. In the retina, this can cause vision loss and blindness [15], while in the spinal cord, it reflects in motor, sensory and autonomic dysfunction. After injury or conditions like ALS, there is progressive atrophy, demyelination and neuronal loss, leading to muscle weakness, paralysis and neuropathic pain [16,17].

For AD, conventional treatments include cholinesterase inhibitors and memantine, which are responsible for symptom attenuation but not for halting disease progression [18].

In PD cases, the gold standard therapy is Levodopa (L-DOPA), a metabolic precursor of dopamine in the brain, which is commonly combined with carbidopa for an increased bioavailability and therapeutic efficacy. Dopamine agonists or MAO-I inhibitors are also an option; however, they do not reverse or slow down the disease progression [19].

Tetrabenazine is one of the first drugs to be used to control involuntary and jerky movements present in HD patients. This treatment is associated with significant side effects and does not halt disease progression. Other drugs, such as deutetrabenazine, a tetrabenazine analogue, have been introduced to achieve similar therapeutic goals, limiting their side effects [18]; however, it is still not ideal.

While some of these conditions are congenital, ageing is considered a significant risk factor for their emergence and progression rate. Moreover, these diseases can progress to a state of dementia, which is expected to affect 150 million people globally by 2050 [2,20].

Although existing, available treatments are mainly supportive and focus on managing the symptoms and improving the patients’ quality of life. Effective therapies remain limited when it comes to the underlying cause of the problem. This is due, in part, to the fact that the precise etiology of many neurological conditions remains unknown, limiting the potential to develop effective targeted therapies [21].

Given these challenges, stem cells have emerged as a promising area of research due to their remarkable therapeutic potential [22].

**Figure 1:**
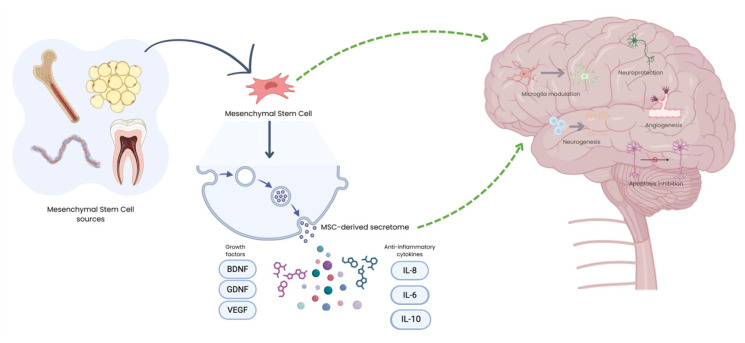
Schematic representation of MSC sources and their therapeutic mechanisms in NDs. Following administration, MSCs exert their effects predominantly through the release of a bioactive secretome, composed of EVs, growth factors and immunomodulatory cytokines. Dark blue curve arrow relates to the obtention of MSCs from its different sources, while green arrows show the mechanisms of action for both MSCs or their secretome. Created in BioRender. Sousa, A. (2026): https://BioRender.com/2qq0i5o (accessed on 16 February 2026).

Mesenchymal stem cells (MSCs) are a promising strategy to restore homeostasis, promote neuroregeneration, and slow disease progression across a broad spectrum of neurodegenerative disorders, since they are capable of healing and reducing inflammation in various tissues [23]. MSCs are multipotent stromal cells widely distributed in the body with good availability and ease of collection, isolation and in vitro culture, capable of being harvested from readily available sources such as autologous and allogeneic bone marrow (BM-MSCs) and adipose tissue (A-MSCs), making them even more clinically desirable. They can also be isolated from human umbilical cord blood (hUCB-MSCs), dental pulp (DPSCs), placenta, amniotic liquid (Amniotic-MSCs), gingival tissue and olfactory mucosa (OM-MSCs) (Figure 1) [24], among other tissues. Despite early evidence of MSC differentiation and cell replacement, recent studies suggest that MSCs differ from other stem cell types by their paracrine effects related to the release of different components derived from their secretome. The soluble fraction of the secretome is enriched in immunomodulatory molecules, cytokines and growth factors, while the vesicular fractions include extracellular vesicles (EVs), which can be categorised according to their size—exosomes or nanovesicles, microvesicles and large EVs (Figure 1) [25,26]. Through this review, the term “MSC-based therapies” covers a broad concept, encompassing both the administration of MSCs themselves as well as their cell-free approaches, based on their secretome—cytokines, growth factors, and EVs.

Using autologous MSCs minimises the risk of immunological rejection, even though allogeneic MSCs are still used, since there is not a significant risk of immunogenicity associated with these cells [24]. MSCs and their products have therefore gained considerable attention, as their use has demonstrated promising results in several areas, including NDs [27]. The secretome includes a variety of molecules such as cytokines, growth factors and microRNAs (miRNAs) which, in most cases, can be packaged into the EVs, allowing for the preservation of the therapeutic characteristics of the MSCs, including angiogenesis promotion and effective tissue repair [4]. EVs are considered promising tools in this type of treatment, as they show an increased potential to cross blood–brain barrier (BBB), thereby enhancing the delivery efficiency and, consequently, their therapeutic effect within the CNS [28]. However, this crossing is influenced by multiple factors, such as cellular origin, size, surface charge, molecular composition and route of administration, remaining partially understood [29,30,31]. These mechanisms of action can be transferred and supported by ND models, validating their treatment potential as MSCs’ neuroregenerative, neuroprotective, and immunomodulatory properties are essential in the context of NDs [4]. The main MSCs’ mechanisms within NDs encompass neuroinflammation reduction, fostering neuron survival in AD, the release of neurotrophic and immunomodulatory factors that support dopaminergic neuron survival and function in PD, and stimulation of neurotropic, immunoregulatory, antioxidant, and antiapoptotic pathways in HD, for example [32]. The combination of multimodal biological efficacy, favourable safety profile, and logistical accessibility positions MSCs and their derived secretome as a desirable source in the quest for effective treatments for NDs.

This systematic review provides a comparative and translational overview of MSC-based therapies across AD, PD, HD and ALS, with a particular emphasis on distinguishing robust preclinical evidence from the still-limited outcomes of early-phase clinical trials, evaluating both cell-based and secretome-derived approaches, and discussing emerging technical strategies aimed at overcoming current translational barriers.

## 2. Data and Methods

### 2.1. Search Strategy and Study Selection

This systematic review was performed according to PRISMA (Preferred Reporting Items for Systematic Reviews and Meta-Analyses) guidelines.

The main goal of this study was to assess the therapeutic potential, methodological approaches and mechanisms of action of MSCs and their derived secretome across different NDs over the past 15 years.

The research involved using the PubMed database, covering the period from 2010 to December 2025. The search query used the following keywords: “Mesenchymal Stem Cells”[MeSH] AND (“Alzheimer Disease”[MeSH] OR “Parkinson Disease”[MeSH] OR “Huntington Disease”[MeSH] OR “Amyotrophic Lateral Sclerosis”[MeSH]), which initially retrieved 485 results. All 485 publications underwent a multistep screening process: at a first point, title and abstract screening was processed, followed by the full-text evaluation.

The inclusion criteria included: (1) studies published in English, (2) full text availability, (3) naïve MSCs or their derived secretome involvement, (4) studies conducted in the context of AD, PD, HD and ALS, and (5) in vivo animal studies or clinical trials.

Exclusion criteria were applied to filter out the studies that did not meet the research goals, such as: (1) in vitro-only studies, (2) review articles and meta-analysis, (3) non-English-language publication, (4) studies unrelated to MSc-based therapies in NDs, (5) studies without full-text access, and (6) studies involving genetically modified, preconditioned or differentiated MSCs, (7) in silico studies, and (8) case reports.

Study selection was conducted systematically, with duplicates managed using Rayyan web application. Each study was screened based on defaulted eligibility criteria. A total of 338 records were excluded after title and abstract screening. The remaining 147 articles were assessed for eligibility via full-text screening, followed by the exclusion of 71 additional studies for not meeting the inclusion criteria. A total of 76 studies were included in this systematic review.

All outcomes specified in the eligibility criteria were sought for each included study. Outcomes were defined a priori and extracted as reported by the study authors. When multiple measures, time points, or analyses were available for the same outcome domain, the results reported in the primary analysis of each study were extracted. If outcomes were reported incompletely or unclearly, this was documented and handled descriptively.

In addition to outcomes, the following variables were extracted where available: study design, stem cells used and their origin. To ensure methodological rigour, the characteristics of MSC sources, administration routes, disease models, and therapeutic outcomes were carefully evaluated. Particular attention was given to distinguishing naïve MSC-based therapies from modified or differentiated MSC approaches to maintain consistency with the defined inclusion criteria.

The assessment was initially conducted by a single reviewer and subsequently verified by all co-authors in order to prevent bias. Any disagreements were resolved through discussion until a consensus was reached. No automation tools were used in the risk of bias assessment.

Due to heterogeneity in study designs, populations, and outcome measures, a meta-analysis was not performed. Instead, findings were synthesised narratively. As no meta-analysis was conducted, statistical methods to assess reporting bias were not applied. However, potential reporting bias was considered qualitatively by examining study protocols where available, comparing reported outcomes with those described in the methods sections, and noting whether expected outcomes were missing from the results. Only published studies were included in this review; therefore, the possibility of publication bias cannot be excluded. The risk of bias within individual studies was assessed qualitatively. Several studies lacked clear reporting of randomisation procedures and blinding, increasing the risk of selection and performance bias. Additional methodological limitations, including small sample sizes and incomplete reporting, were also identified in some studies. A formal GRADE assessment was not conducted. However, the overall certainty of the evidence for each outcome was judged based on study design, consistency of findings, methodological limitations and sample sizes.

**Figure 2:**
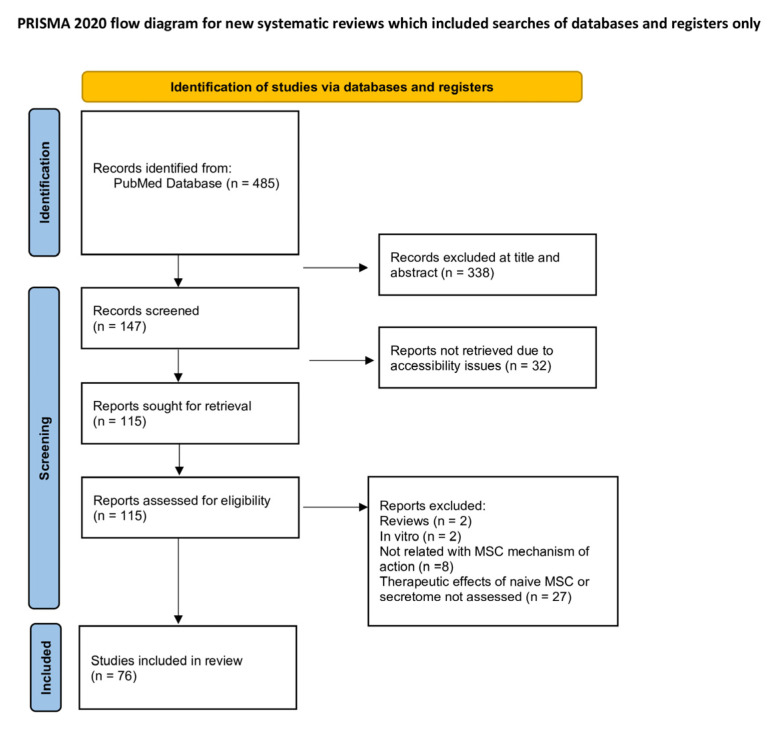
PRISMA Flow Diagram that summarises the selection process.

The selection process is summarised in Figure 2.

### 2.2. Protocol Registration

This systematic review was prospectively registered in the Open Science Framework (OSF) registries with the URL https://osf.io/trz4g (accessed on 16 February 2026) with DOI: 10.17605/OSF.IO/ABHGD.

## 3. MSC’s Therapies for Specific Neurological Disorders

Recent studies have been conducted to find effective therapeutic solutions for the treatment of ND using stem cells, due to their self-renewal, self-replicating, regenerative and differentiation capabilities [33].

### 3.1. Alzheimer’s Disease (AD)

**Figure 3:**
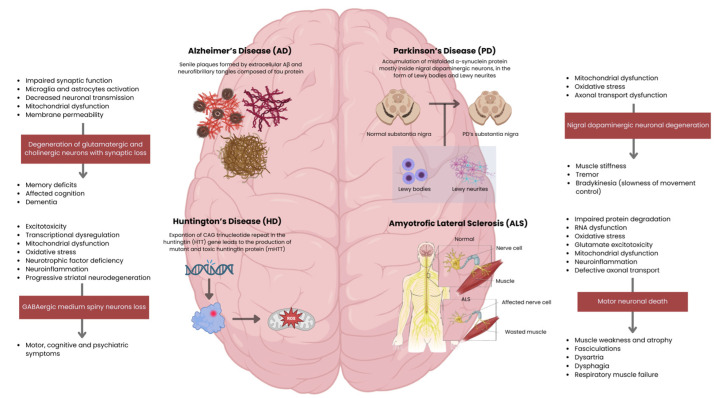
Pathophysiological mechanisms and clinical hallmarks of major neurodegenerative diseases. Side panels delineate the progression from cellular dysfunction to specific neuronal loss and subsequent clinical manifestations. Created in BioRender. Sousa, A. (2026): https://BioRender.com/92g181j (accessed on 16 February 2026).

AD is one of the most prevalent types of NDs, mostly affecting the ageing population. Clinically, AD is associated with dementia, characterised by a progressive loss of memory and impairment of one or more cognitive domains. Degeneration primarily affects hippocampal and cortical neurons, including glutamatergic and cholinergic populations, leading to memory loss and cognitive decline (Figure 3) [34,35].

Current evidence from pre-clinical and clinical studies suggests the amyloid-β peptide (Aβ and Tau protein) as the central contributor to AD pathophysiology [36]. Their deposition into insoluble states in determined histopathological brain structures—senile plaques formed by extracellular Aβ (between neuronal connections) and neurofibrillary tangles (NFTs) composed of intracellular Tau—is a mark of more advanced disease. As Aβ aggregates into insoluble plaques between neurons, synaptic communication is compromised by neurotransmission impairment [37]. Beyond this mechanical disruption, Aβ plaques act as potent activators of the innate immune system. Microglia and astrocytes recognise them as a danger signal and attempt to clear the plaques, but their sustained activation leads to chronic neuroinflammation. This inflammatory state exacerbates neuronal damage through the release of inflammatory cytokines, ROS, and other neurotoxic mediators (Figure 3). Thus, Aβ accumulation contributes both to synaptic failure and to a self-perpetuating inflammatory cycle that accelerates neurodegeneration in AD [38]. Moreover, soluble and scattered forms of Aβ and Tau—especially their oligomer forms—can also be responsible for significant neurotoxic effects [39]. These small and mobile species can diffuse through the extracellular space and cross-neuronal membranes and synapses [40]. Because they are soluble and not stagnant, these molecules can interact directly with neurons and synapses, causing synaptic dysfunction through changes in neurotransmitter release [37], disruption of neuronal membranes by increasing their permeability, mitochondrial dysfunction within neurons [41], and neuroinflammation due to the circulation of foreign molecules detected by microglia and astrocytes, enhancing the inflammatory response (Figure 3) [42].

Current therapies are based on symptomatic relief rather than addressing the root cause of this neurological disorder. Attempted therapeutics aiming at the reduction in Aβ levels have proved unsuccessful. Similarly, Tau-based clinical trials have not yet yielded positive results [43].

Preclinical studies have shown that MSCs and their derivatives can promote beneficial effects in different animal models of AD. Preclinical studies have consistently demonstrated that mesenchymal stromal cells (MSCs) and their derivatives exert beneficial effects in different experimental models of Alzheimer’s disease (AD). Studies employing various MSC sources—BM-MSCs, A-MSCs, hUCB-MSCs, human amniotic-derived MSCs (hAmniotic-MSCs), OM-MSCs and their extracellular vesicles (EVs)—reported improvements across multiple pathological and functional hallmarks of AD in both acute and transgenic murine models [44,45,46,47,48].

In mice and rat models such as Aβ1-42 [44,49,50], APP/PS1 [47,51,52,53,54,55,56,57,58], 5xFAD [45,59,60], Tg2576/APPswe and 3xTg mice [61,62], and other non-transgenic AD models [57,63,64,65,66], MSC-based therapies administered through different routes (intrahippocampal, intracerebroventricular, intranasal, or intravenous) were associated with reduced amyloid-beta burden and decreased plaque formation [45,46,47,51,52,55,61,64] and modulation of neuroinflammatory responses [44,48,60,66]. Several studies also showed that MSC treatment promoted a shift in microglia toward an anti-inflammatory phenotype and reduced astrocyte activation, suggesting a broader impact on neuroinflammation beyond amyloid pathology [51,56,61,64].

Beyond amyloid-related effects, a subset of studies using models that also develop Tau pathology reported reductions in Tau hyperphosphorylation following MSC administration. These findings support the potential of MSCs to target multiple AD hallmarks rather than acting solely on Aβ deposition [53,61].

Improvements in spatial learning and memory performance [46,48,49,54,61,62,63,64], and anxiety-related behaviours [60] were reported in both acute Aβ-injection models and chronic transgenic models after treatment with MSCs or MSC-derived exosomes. Intranasal delivery of MSC-derived small EVs showed the ability to cross the blood–brain barrier and reach the central nervous system while improving cognitive performance, highlighting a non-invasive administration route with translational potential [45].

This evidence suggests that the therapeutic benefits of MSCs are largely mediated through paracrine effects rather than long-term cell engraftment. Most of the IV-administered MSCs become trapped in peripheral organs, reinforcing the idea that secreted factors, immunomodulation, and EVs play a central role in neuroprotection, synaptic preservation, and neurogenesis [48,61].

Clinical research has progressed from early safety trials to more advanced studies. A Phase I trial using repeated intracerebroventricular injections of hUCB-MSCs in AD patients found the procedure to be well-tolerated, with only mild, transient side effects. However, limitations were observed regarding dosage, since it was adapted from previous mouse studies [67]. Additionally, a Phase 2a randomised controlled trial of allogeneic BM-MSC therapy, Laromestrocel, demonstrated safety and indications of efficacy in mild AD. Among 49 patients, the treatment met its first primary safety endpoint, and efficacy analysis showed that the combined treatment groups slowed brain and hippocampal atrophy and improved clinical scores. This structural preservation was correlated with improved cognitive performance. This trial included a small sample size with a relatively short 39-week duration [68].

Overall, the strength of evidence derived from preclinical models has not yet been obtained from early-phase clinical trials. While animal studies consistently demonstrate that MSCs and their derivatives can ameliorate cognitive deficits and reduce key pathological hallmarks of AD, these findings are generated under highly controlled experimental conditions and may not fully translate to the human disease. In contrast, current clinical evidence is still limited to Phase I and early Phase II trials, which are primarily designed to assess safety and feasibility rather than clinical efficacy, with small-sized cohorts. Although preliminary signals of cognitive stabilisation and reduced brain atrophy are encouraging, they remain exploratory and insufficient to draw definitive conclusions regarding therapeutic effectiveness. Therefore, the promising outcomes observed in preclinical studies should be interpreted with caution, underscoring the need for larger, well-powered, and long-term clinical trials to validate the translatability of MSC-based therapies for AD.

### 3.2. Parkinson’s Disease (PD)

PD is the second most common ND, primarily affecting the ageing population. The pathophysiology associated with this disease is related to the accumulation of misfolded α-synuclein protein inside neurons, in the form of Lewy bodies (LBs) and Lewy neurites [69]. PD is pathologically characterised by the loss of nigrostriatal dopaminergic innervation, although neurodegeneration is not limited to nigral dopaminergic neurons, which are responsible for producing dopamine in the substantia nigra and sending it to the striatum, enabling normal movement control (Figure 3). Such a widespread pathology makes PD a very heterogeneous disorder, and a reliable diagnostic test is not yet available. Clinical criteria, however, can only lead to a diagnosis of probable PD, while a definitive diagnosis requires histopathological assessment, with the identification of α-synuclein-containing LBs or Lewy neurites, suggesting that α-synuclein plays a central role in PD pathogenesis [70].

Preclinical evidence from toxin-based models such as 6-OHDA [71,72,73,74,75,76], rotenone [77] and MPTP [78,79], as well as genetic and α-synuclein-based models [80,81,82,83], indicates that MSC-based therapies can attenuate dopaminergic degeneration, reduce neuroinflammation, and improve motor performance across different PD models and routes of administration.

Both direct MSC transplantation and administration of their secretome have shown therapeutic efficacy, supporting the notion that much of the benefit is mediated by paracrine mechanisms rather than long-term cell engraftment [72,80,84,85]. A comparative study revealed that intracranial delivery of hBM-MSC secretome can improve fine motor performance when compared to Levodopa administration [84].

In models of α-synuclein pathology, MSC-derived secretome reduced nigral α-synuclein accumulation, protected dopaminergic neurons in the substantia nigra and striatum and modulated microglial reactivity [80,82].

Across toxin-induced models, MSCs from different sources—including BM-MSCs, UC-MSCs, Amniotic-MSCs and Gingival-MSC—improved motor function [71,72,75,77,79,82], restored dopamine levels and reduced oxidative stress [71,77], and limited neuronal and glial death. These effects were observed after intracerebral, intrastriatal, intranasal or intravenous administration, through immunomodulation and release of neurotrophic factors such as BDNF and GDNF [76,79].

Molecules found in the secretome multitarget disease-modifying effects on PD hallmarks, including α-synuclein aggregation in LBs and Lewy neurites (Figure 3), by neurotrophic factors secretion and EVs that cross the BBB, support dopaminergic neuron survival, and improve motor behaviour in toxin and α-synuclein-based PD models [86,87].

Clinical trials with allogeneic BM-MSCs, such as Schiess et al.’s [88] single-centre, open-label, dose-escalation Phase I clinical trial involving 20 patients with mild-to-moderate PD, aimed to assess the safety and tolerability of a single cell intravenous infusion. The study met its primary safety endpoint, demonstrating that the infusion was safe, well-tolerated, and non-immunogenic.

In terms of exploratory efficacy, the highest dose group showed the most significant clinical benefit, sustaining a reduction in the UPDRS motor score at 52 weeks. Biologically, this clinical improvement was accompanied by a potent immunomodulatory effect, characterised by a decrease in peripheral inflammatory markers and an increase in BDNF. The authors acknowledge several limitations inherent to early-stage trials: a small sample size (*n* = 20) and an open-label design without a placebo control group. Additionally, the study did not include genetic testing or subtyping as part of the dose assignment, which ignores potential phenotypic heterogeneity that could influence treatment response [88].

Building on their Phase I results, Schiess et al. [89] conducted a Phase II, randomised, double-blind, placebo-controlled trial with 45 patients to evaluate the efficacy of repeated intravenous infusions of allogeneic BM-MSCs. Participants were randomised to receive either three active infusions, two active infusions (preceded by one placebo), or three placebo infusions, administered at 18-week intervals over 36 weeks. The study met its primary endpoint and confirmed its safety profile, with no severe adverse events or immunogenicity reported.

Despite the positive outcome for the high-frequency group, the study revealed a confusing discrepancy: the group receiving two infusions performed significantly worse than the placebo group, showing minimal improvement. Furthermore, a substantial placebo effect was observed in the control group, complicating the interpretation of the results. Other limitations include the single-centre nature of the trial, limiting generalisability, and a small sample size [89].

The available evidence highlights a clear distinction between preclinical observations and clinical outcomes in MSC-based approaches for PD. Experimental models demonstrate that MSC secretome and EVs can modulate key pathological mechanisms, including neuroinflammation, α-synuclein accumulation and dopaminergic neuronal vulnerability. However, translation of these findings to the clinical setting remains limited. Human studies to date are restricted to early-phase trials with small sample sizes, heterogeneous methodologies and primary endpoints focused on safety rather than efficacy. Reported clinical improvements are variable and may reflect transient or non-specific effects, given the absence of adequate controls, standardised dosing regimens and long-term follow-up.

### 3.3. Huntington’s Disease (HD)

HD is an autosomal dominant ND caused by an expanded CAG trinucleotide repeat in the huntingtin (HTT) gene, leading to the production of mutant and toxic huntingtin protein (mHTT) [90]. mHTT primarily affects medium spiny GABAergic neurons in the striatum (caudate nucleus and putamen), leading to their progressive degeneration. Cortical neurons may also be affected as the disease progresses, contributing to cognitive decline and psychiatric symptoms [91].

The pathophysiology of HD involves multiple mechanisms: excitotoxicity, transcriptional dysregulation, mitochondrial dysfunction, oxidative stress, impaired proteostasis and reduced availability of neurotrophic factors. Additionally, astrocyte dysfunction and neuroinflammation contribute to disease progression, resulting in the characteristic motor, cognitive, and psychiatric symptoms (Figure 3) [92].

Although mutant huntingtin (mHTT) is the primary genetic driver of HD, current MSC-based strategies do not aim to directly suppress or eliminate mHTT expression. Instead, they are designed to mitigate downstream pathogenic processes triggered by mHTT, including neurotrophic factor deficiency, neuroinflammation, mitochondrial dysfunction, and progressive striatal neurodegeneration [32,93].

Preclinical studies in transgenic models of HD, particularly R6/2 mice [94,95,96,97,98] and 51 CAG rats [99], indicate that MSC-based therapies can exert neuroprotective and functional benefits despite not reversing the underlying genetic cause of the disease.

Across different studies, MSCs were associated with preservation of striatal structure alongside improvements in multiple motor outcomes [96,97] such as motor coordination [95]. Effects on cognitive performance were generally limited or absent [94], and considerable heterogeneity was observed in motor coordination tests, reflecting differences in animal models, MSC sources, routes of administration, and timing of transplantation [97].

MSC therapy in HD models reduced apoptosis and neuroinflammation while upregulating neurotrophic support, particularly Brain-derived Neurotrophic Factor (BDNF) [97].

Local and non-invasive delivery strategies like intrastriatal transplantation of MSCs improved motor deficits, increased BDNF levels and delayed disease progression in R6/2 mice [97], while intranasal administration targeted cells to the striatum, prolonging survival and ameliorating dopaminergic and inflammatory alterations [98].

This evidence supports a paracrine mechanism of action for MSCs in HD, involving neuroprotection, immunomodulation, and enhancement of neurotrophic signalling rather than neuronal replacement.

Evidence for MSC-based interventions in HD remains confined to preclinical studies. While animal models show that MSCs can modulate neuroinflammation, apoptosis and neurotrophic support, translating these effects to humans has not yet been demonstrated. Clinical data are limited to observational trials focused on safety rather than efficacy. At present, MSC-based approaches in HD should therefore be considered experimental, requiring further rigorous evaluation before clinical relevance can be established.

This gap between robust preclinical efficacy and the absence of validated clinical evidence underscores the translational challenges in HD, including limitations of rodent models, uncertainties regarding optimal delivery strategies, dosing, and long-term safety, and the need for large-animal models to better understand disease progression before broad clinical implementation [100].

### 3.4. Amyotrophic Lateral Sclerosis (ALS)

ALS is a progressive neurodegenerative disorder characterised by the selective loss of motor neurons that normally control voluntary muscle contraction, typically leading to death within 2 to 4 years after symptom onset. Approximately 10% of ALS patients have a family history consistent with an autosomal dominant inheritance pattern. The remaining 90% of cases are sporadic, with no affected family members, and are classified as sporadic ALS (SALS) [101].

Although the precise mechanisms underlying motor neuron degeneration remain unclear, studies using SOD1 mutant models and other ALS-associated genes have implicated multiple molecular pathways. These include impaired protein degradation, RNA dysregulation, oxidative stress, glutamate excitotoxicity, mitochondrial dysfunction, aberrant neuroinflammation, and defective axonal transport, all contributing to motor neuron death [102,103]. Clinically, this neuronal loss manifests as progressive muscle weakness, atrophy and fasciculations, reflecting the combined involvement of lower and upper motor neurons (Figure 3) [104]. As the disease advances, patients develop dysarthria, dysphagia, and respiratory muscle failure, which ultimately represent the main cause of death in ALS [105]. Sensory functions are typically preserved, although a subset of patients may also exhibit cognitive and behavioural impairment, associated with frontotemporal dysfunction [106].

A systematic review and meta-analysis of preclinical ALS studies in transgenic mice and other rodent models found that MSC transplantation significantly delayed onset, slowed clinical progression and extended survival. Reviews emphasise that benefits are largely mediated by paracrine neurotrophic and anti-inflammatory actions, rather than neuron replacement [107,108].

MSCs secrete multiple neurotrophic and growth factors—BDNF, Glial cell line-Derived Neurotrophic Factor (GDNF), and Vascular Endothelial Growth Factor (VEGF) [107]—and anti-inflammatory cytokines, which collectively modulate microglial and T-cell activation, and protect motor neurons from excitotoxicity and oxidative stress [109]. These effects contribute to the preservation of motor function and neuronal survival in ALS animal models.

Preclinical studies were conducted using SOD1 G93A mice or rat models of ALS, showing that MSC-based therapies can delay motor decline or improve motor performance [110,111,112,113] and, in many cases, prolong survival [111,113,114,115,116,117]. These benefits were observed after different routes of administration—intrathecal, intracerebroventricular, intranasal and IV delivery.

These therapies act mainly through immunomodulation and neuroprotection, reducing neuroinflammation, modulating microglia [110,112,115,116,118,119] and promoting spinal cord vascular repair rather than replacing neurons [120]. Despite promising results, some studies reported limited effects on muscle denervation [113,114,121] and short-term cell persistence [117,118], highlighting the need for optimised cell sources and dosing strategies before clinical translation.

Early-phase clinical trials focused on safety, feasibility and preliminary function outcomes confirmed that intraspinal autologous BM-MSC delivery had no serious transplant-related toxicity or tumour formation over months to years of follow-up. Also, intrathecal (IT) BM-MSC in 26 patients was safe and associated with transient slowing of ALS Functional Rating Scale (ALSFRS) decline and stabilisation of forced vital capacity (FVC) in many patients for several months [122].

Petrou et al. [123] conducted a single-centre, open-label Phase II clinical trial involving 20 patients to evaluate the safety and efficacy of repeated intrathecal injections of autologous BM-MSCs. The treatment proved to be safe and well-tolerated, with no serious adverse events directly related to the MSC administration.

In terms of efficacy, the study reported a significant reduction in the rate of disease progression; many of the patients were classified as responders, demonstrating a greater improvement in the slope of their ALSFRS-R score compared to the pre-treatment run-in period, with some patients showing actual clinical improvement. Notably, the study highlighted that the clinical benefit appeared to be transient and correlated with the frequency of administration, as shorter time intervals between injections yielded more robust responses than longer intervals, supporting the necessity of repeated dosing cycles to sustain therapeutic effects [123].

Repeated IT autologous BM-MSC injections, delivered in three doses, at one-month intervals, were also safe, showing ALSFRS stabilisation and improved FVC over a three-month period. Similar repeated IT protocols showed acceptable safety over 12 months. The lack of post-mortem material restricts the drawing of conclusions regarding the fate of MSCs after injections [124].

In general, MSC-based approaches for ALS show some divergences between biological effects and clinical outcomes. Preclinical and clinical studies consistently demonstrate acceptable safety and modulation of inflammatory and neurotrophic biomarkers. However, effects on disease onset and progression, or survival, remain inconsistent. Even in more advanced clinical trials, observed functional benefits are modest, subgroup-restricted, and underpowered, precluding firm conclusions regarding disease-modifying efficacy in humans.

The relevant preclinical evidence across AD, PD, HD and ALS is summarised in Table 1, while the main clinical findings in AD, PD and ALS are presented in Table 2.

Overall, a moderate risk of bias due to missing results was considered across all disease-specific syntheses. Although some studies reported limited efficacy or methodological weaknesses, the predominance of positive or partially positive findings suggests that studies with neutral or negative outcomes may still be underrepresented in the literature.

## 4. Discussion

Across multiple NDs, MSCs have consistently demonstrated a favourable safety profile and a broad spectrum of biological activities that target key pathogenic mechanisms shared by AD, PD, HD and ALS. Rather than acting through direct neuronal replacement, MSCs exert their effects predominantly via paracrine mechanisms, including the secretion of neurotrophic factors, anti-inflammatory cytokines, and EVs [127]. This multimodal mechanism of action represents a major advantage in diseases characterised by complex, multifactorial and not fully understood pathophysiology.

Nevertheless, translating these biological properties into robust and sustained clinical benefit has proven challenging. One of the main limitations associated with MSC administration is their limited survival [128], engraftment, and long-term persistence within the CNS. Following transplantation, MSCs are exposed to a hostile microenvironment marked by oxidative stress, inflammation, and ongoing neurodegeneration, which likely restricts their therapeutic durability. Moreover, systemically administered MSCs show inefficient homing to the brain and spinal cord, with significant cell trapping in peripheral organs and restricted passage across the BBB [129,130].

Another important challenge lies in the intrinsic heterogeneity of MSC treatments [131]. Differences in tissue source, donor-related characteristics, isolation methods, and culture conditions contribute substantially to variability in MSC secretory profiles and therapeutic potency, thereby limiting direct comparability across studies and likely contributing to inconsistent clinical outcomes. Distinct donor biological backgrounds, and even from different species in preclinical settings, profoundly influence MSCs’ secretory (EVs and other molecules) profiles and composition. These differences are further amplified by variability in isolation and expansion strategies, passage number, and cell culture protocols.

Notably, the fact that MSCs derive from multiple tissue sources introduces significant biological heterogeneity across trials within each ND and further complicates the translational landscape. Despite this variability, clinical studies frequently interpret outcomes collectively, without adequately accounting for source-dependent functional differences. As a result, both MSC-based and MSC-derived secretome therapies represent highly heterogeneous biological products rather than standardised interventions [132].

The source of translational uncertainty also arises from the substantial variability in dosing strategies—including substantial variation in the number of administered cells or secretome concentration—employed in preclinical studies. Experimental models of ND use divergent doses from different sources of MSCs or MSC-derived secretome, administered by different routes and dosing frequencies, often without systematic evaluation of the dose–response relationship.

This methodological heterogeneity limits the extrapolation of preclinical efficacy data to the clinical setting, as the optimal dose of cells or secretome required to overcome well-known barriers, such as inefficient homing, peripheral entrapment, and limited bioavailability in the CNS, remains unknown. Consequently, clinical dosing regimens are often selected empirically, contributing to a disconnect between preclinical dosing paradigms and clinical trial design. Thus, the modest and variable efficacy observed in human studies underscores the need for standardised preclinical frameworks capable of finding the optimal dose for clinical translation [133]. Without harmonised criteria for MSCs source choice, culture protocols and dosage methodology, it remains challenging to support comparable results from MSC-derived therapies. As a result, establishing standardised protocols that guarantee viable performances is essential and desirable to design robust and scalable clinical trials capable of reaching later-phase developments.

The clinical translation of MSCs and MSCs-derived secretome is dependent on regulatory success, which requires clear potency assays. However, the design of meaningful potency assays is further complicated by the incomplete understanding of MSCs’ dominant mechanisms, as they do not represent a mechanistically uniform therapy: their biological activity is highly context-dependent, reinforcing the MSCs’ heterogeneous profile. Therefore, the relative contribution of immunomodulatory, neurotrophic, or EV-mediated effects may vary not only between MSC products but also across different NDs. In this context, potency assays based on single functional readouts risk oversimplifying MSC activity and may fail to predict clinical efficacy. Without a clear understanding of which mechanisms are critical for modulating disease pathophysiology and specific pathways, it remains challenging to define which assays are relevant and broadly applicable.

Despite these challenges, the overall clinical experience to date remains encouraging: MSC-based therapies have repeatedly shown reliable safety profiles across intrathecal, intravenous, and intracerebral administration, with minimal serious adverse events reported in early-phase clinical trials [134,135]. Even in trials where primary clinical endpoints were not met, secondary outcomes and biomarker analyses frequently revealed biologically meaningful effects, such as modulation of neuroinflammation, preservation of neurotrophic support, and slowing of functional decline in selected patient subgroups [136]. These findings suggest that the modest clinical efficacy observed thus far may reflect suboptimal patient stratification, dosing strategies, or disease stage selection, rather than a fundamental lack of therapeutic relevance.

## 5. Conclusions and Future Directions

This review emphasises the progress in MSC therapy for NDs, including AD, PD, HD and ALS. Despite the remarkable advancements, particularly in early-phase clinical trials, the scarcity of late-stage trials highlights the necessity for further investigation.

One of the most consistent barriers limiting MSC efficacy in NDs relies on their poor survival, inefficient homing to the CNS and peripheral entrapment following systemic administration. Transplantation exposes MSCs to a hostile environment, and their intravenous administration restricts their crossing through the BBB, reducing their bioavailability. To overcome these limitations, preconditioning strategies have emerged as rational approaches to enhance MSC resilience in NDs’ inflammatory environment. Priming can possibly increase neuroprotective and anti-inflammatory factors, improving functional persistence, enhancing MSC survival [137]. Genetic modifications with survival genes and homing receptors have shown potential in improving the issues related to poor CNS homing and MSC survival, as they can potentially enhance resistance to hypoxia while improving targeted migration to lesion sites [138]. Also, shifting from cell-based to secretome therapies may bypass homing and survival constraints, allowing the therapeutic effects to be sustained or even amplified regardless of cell persistence.

Another difficulty associated with clinical translation is the intrinsic heterogeneity of MSC-based therapies. This variability is further amplified by the manufacturing and culture protocols, giving rise to highly heterogeneous biological products, rather than standardised interventions. To mitigate this challenge, standardisation-oriented frameworks are essential. Efficacy is maximised by matching the cell source (as different sources produce MSCs with different capacities and characteristics) to the biological specific requirement, as a precise “fit-for-purpose” selection. Studies have also shown that EVs from different tissues inherit distinct homing patterns and protein cargos. By systematically profiling and matching these intrinsic strengths to the clinical disease or pathophysiology, researchers can establish a “rational selection” that resolves the uncertainty related to the traditional model used in preclinical studies based on MSC application indiscriminately across different disease contexts and sites [139].

The lack of a well-defined dose–response relationship corresponds to another major limitation of MSC-based therapies. Preclinical studies operate with divergent and heterogeneous dosages, often without systematic evaluation of therapeutic thresholds. Since no optimisation of the administered dose was conducted in the preclinical investigation, dosing strategies are based on empirical extrapolation rather than robust preclinical optimisation. Addressing this limitation requires the standardisation of preclinical dosing frameworks, including rigorous dose–response studies to improve translational reliability [140].

In addition, a further translational problem lies in the insufficient implementation of standardised potency assays and regulatory frameworks, capable of capturing the functional heterogeneity of MSCs. Unlike small molecules, MSCs exert therapeutic effects through multifaceted mechanisms, including immunomodulation, neuroprotection and paracrine signalling. In the field of NDs, such assays remain poor and inconsistently applied. The lack of regulatory and development frameworks has resulted in an empirical and often indiscriminate use of heterogeneous MSC products across different disease contexts [141]. Frameworks such as mechanism-driven or fit-for-purpose models are critical to integrate cell source selection, manufacturing conditions, dosing strategies and the rational choice of potency assays. Without this coordinated approach, MSC-based interventions continue to be affected by suboptimal translational predictability.

Future research should focus on broadening clinical trials to encompass more NDs, also addressing the issues of scalability and standardisation of clinical translational protocols. The rising interest in EV-based therapeutics, genetic engineering and MSC preconditioning, alongside technological progress and an enhanced comprehension of their therapeutic potential, is anticipated to refine next-generation treatment techniques for NDs. These advancements may facilitate more accurate, efficient, and less invasive procedures, thereby enhancing patient outcomes and clinical translation in these NDs.

## Figures and Tables

**Table 1:** Preclinical investigation in animal models supporting MSC-based therapies in AD, PD, HD and ALS.

Reference	Animal Model	Cell Source/Product	Route	Main Outcomes
[44]	AD Aβ_1-42_ rats	A-MSCs	Intrahippocampal	Improved memory and learning; reduced inflammation
[63]	AD OBE-induced mice	Human foetal MSCs	IV/Itracerebroventricular	Safe; both routes improved spatial memory
[51]	AD APP/PS1 mice	mBM-MSCs/NSCs (comparison)	Intrahippocampal	Reduced number of Aβ plaques; promoted anti-inflammatory microglia; failed to reduce hyperlocomotion
[45]	AD 5XFAD mice	hBM-MSCs Small EVs	IN	Improved cognition; reduced Aβ plaque number; reduced inflammation
[52]	AD APP/PS1 mice	BM-MSCs EVs	Intracerebral	Reduced size and number of Aβ plaques; prevent plaque formation
[46]	AD Aβ_1-42_ mice	mOM-MSCs exosomes	IV	Improved spatial learning; reduced Aβ levels; neuroprotection
[59]	AD 5XFAD mice	hUC-MSCs	Intracerebroventricular	Restored synapse density and specific receptors related to synapse maintenance
[47]	AD APP/PS1 mice	hUCB-MSCs	Intrahippocampal	Reduced Aβ plaques
[48]	AD Tg2576 APPswe mice	hAmniotic-MSCs	IV	Improved spatial learning and memory; immune modulation
[53]	AD APP/PS1 mice	hUCB-MSCs	Intrahippocampal	Switched microglia to an anti-inflammatory state; reduced Aβ plaques and Tau hyperphosphorilation; increased spatial learning and memory
[64]	AD Abeta mice	hUCB-MSCs	Intrahippocampal	Neuroprotection; reduced microglial activation; restored learning and memory functions
[54]	AD APP/PS1 mice	mBM-MSCs	Intrahippocampal	Reduced Aβ plaques; improved spatial learning
[65]	AD STZ mice	mBM-MSCs exosomes	IV/Intracerebroventricular	Restored BDNF levels and synaptic proteins
[55]	AD APP/PS1 mice	mBM-MSCs	Intrahippocampal	Increased memory; reduced Aβ plaques
[56]	AD APP/PS1 mice	mA-MSCs	Intrahippocampal	Microglia modulation; reduced amyloid burden; restored spatial memory
[60]	AD 5xFAD mice	mBM-MSCs	Lateral ventricle/Hippocampal/Combined	Reduced anxiety levels; reduced inflammation; amyloid levels modulation
[61]	AD 3xTg mice	mBM-MSCs	IV	Reduced Tau hyperphosphorilation; not significant decrease in Aβ plaques; reduced microglia and astrocyte activation
[49]	AD Aβ_1-42_ mice	hMSCs	IV	Neurogenesis; improved memory performance
[62]	AD 3xTg mice	mBM-MSCs	IV	Decreased number of plaques; increased spatial memory
[125]	AD AlCl3-induced rats	mBM-MSCs exosomes	IV	Improved spatial memory; in healthy rats, exosomes impaired spatial memory
[57]	AC ICR and APP/PS1 mice	hBM-MSCs	IV	Promoted fusion between autophagosomes and lysosomes; clearance of Abeta; reduced neuronal death
[50]	AD Aβ_1-42_ mice	hOlfatory Ecto-MSCs	IN/Intrahippocampal	Safe; improved memory; reduced Aβ plaques
[58]	AD APP/PS1 mice	hAmniotic-MSCs	IV	Angiogenesis; cells crossed BBB, entering the brain
[66]	AD ICR mice	hPlacenta-MSCs	IV	Reduced pro-inflammatory cytokines; neurogenesis
[71]	PD 6-OHDA rats	hBM-MSCs	Instrastriatal	Reduced motor symptoms; neuroprotection through the released factors
[72]	PD 6-OHDA rats	hUC-MSCs exosomes	IV	Improved motor function; neuroprotection
[73]	PD 6-OHDA mice	hUC-MSCs	IV	Systemic oxidative stress reduction; cell entrapment in the spleen
[74]	PD 6-OHDA rats	mBM-MSCs	IN	Improved fine motor control; restored dopamine levels; reduced inflammation
[77]	PD Rotenone rats	mBM-MSCs	IV/Intrastriatal	Improved motor function, dopamine levels and reduced inflammation
[78]	PD MPTP rats	mBM-MSC	Intracisternal	Prevented neuronal and glial death
[85]	PD MPTP mice	hUC-MSCs exosomes	IN	Improved non-motor symptoms; improved motor coordination; neuroprotection
[81]	PD AAV-ER-α-Syn mice	hBM-MSCs	IV	Accumulated α-synuclein clearance; neuroprotection; improved motor symptoms
[75]	PD 6-OHDA rats	hGengival-MSCs	IV	Safe; improved motor symptoms; neuroprotection
[80]	PD AAV-A53T-α-Syn rats	hBM-MSCs secretome	Intracerebral	Reduced the accumulation of α-synuclein; modulated microglia; improved fine motor control
[82]	PD α-Syn injection mice	hMSCs	IV	Inhibited cell-to-cell spread of toxic α-synuclein; neuroprotection; improved motor function
[83]	PD AVV-mediated α-Syn overexpression	hMSCs	IV	Autophagosomes ans lysossomes fusion to clear α-synuclein
[79]	PD MPTP mice	hA-MSCs	IV	Safe; restored levels of BDNF and GDNF in the striatum; improved motor function
[76]	PD 6-OHDA rats	hA-MSCs	Intranigral	Neurogenesis; cells expressed BDNF and endothelial markers
[84]	PD 6-OHDA rats	hBM-MSCs secretome	Intracranial	Improved fine motor coordination compared to Levodopa-treated animals
[94]	HD R6/2 mice	mUC-MSCs	Intrastriatal	Reduced brain atrophy and metabolic loss; transient memory improvement; no long-term motor recovery
[95]	HD R6/2 mice	hAmniotic-MSCs secretome	Intraperitoneal	Improved coordination; reduced brain shrinkage; reduced inflammation
[96]	HD R6/2 mice	hBM-MSCs	Intrastriatal	Recruitment of the host’s own MSCs; angiogenesis; increased lifespan; reduced motor deficits
[99]	HD 51 CAG transgenic rats	mBM-MSCs	Intrastriatal	Restored behaviour; immunomodulation
[97]	HD R6/2 mice	mBM-MSCs	Intrastriatal	Increased levels of BDNF; delayed motor decline
[98]	HD R6/2 mice	mBM-MSCs	IN	Extended survival; ameliorated circadian rhythm
[110]	ALS SOD1 G93A mice	hBM-MSCs	Intrathecal	Delayed motor decline; neuroprotection; reduced inflammation
[114]	ALS SOD1 G93A mice	hBM-MSCs	Intrathecal	Prolonged lifespan; neuroprotection; short-term cell survival
[111]	ALS SOD1 G93A rats	mBM-MSCs	IV/Intraspinal	Prolonged lifespan; improved motor activity; neuroprotection
[112]	ALS SOD1 G93A mice	hBM-MSCs (“Responder” vs. “Non-responder” cells)	Instracisternal	Responder cells lived longer; improved motor performance; reduced inflammation; neuroprotection
[120]	ALS SOD1 G93A rats	mBM-MSCs	IV	Spinal cord vascular repair; neuroprotection
[118]	ALS SOD1 G93A mice	mA-MSCs	IV	Glial modulation; delayed motor deterioration; not extended lifespan
[113]	ALS SOD1 G93A rats	hBM-SMCs (cells vs. secretome)	Intrathecal/Intramuscular	Increased lifespan; improved motor symptoms; not significantly reduced necropt biomarkers
[119]	ALS SOD1 G93A mice	hUC-MSCs	Intracerebroventricular	Shifted cytokines to an anti-inflammatory state; did not prevent muscle denervation or extend survival
[115]	ALS SOD1 G93A mice	hAmniotic-MSCs	IV	Extended lifespan; improved motor performance; decreased inflammation
[116]	ALS SOD1 G93A mice	UC-MSCs secretome	IV	Extended lifespan; reduced inflammation; microglia modulation
[126]	ALS SOD1 G93A mice	hUC-MSCs	IV	Neuroprotection; extended lifespan; delayed motor decline
[121]	ALS SOD1 G93A mice	A-MSCs EVs	IN	Preserved structure of neuromuscular junctions; improved motor performance; did not extend overall lifespan
[117]	ALS SOD1 G93A mice	mBM-MSCs	IV	Prolonged lifespan; downregulated oxidative stress markers

**Table 2:** Clinical trials supporting MSC-based therapies in AD, PD and ALS.

Disease	Reference	Trial Phase	Cell Source/Product	Administration Route	Dosage	Administration Frequency	Outcomes	Limitations
AD	[67]	Phase I	hUCB-MSCs	Intracerebroventricular	2 groups: Low: 1 × 10^7^ cells High: 3 × 10^7^ cells/2 mL	3 repeated injections at 4-week intervals	Safe, feasible and well-tolerated; no structural abnormalities	Injection dose based on mouse studies; transient fever and mild symptoms following MSC administration
AD	[68]	Phase IIa	Allogeneic BM-MSCs (laromestrocel)	IV	2 doses: 25 × 10^6^ cells 100 × 10^6^ cells	(G1: placebo, ×4: G2: 25 M × 1 + placebox3; G3: 25 × 4; G4: 100 × 4) monthly	Safe and well-tolerated; clinical efficacy (secondary and exploratory outcomes); cognitive, functional and structural improvements	Small sample size; short study duration
PD	[88]	Open-label, Phase I	Allogeneic BM-MSCs	IV	1, 3, 6 or 10 × 10^6^ cells/kg	Single dose	Safe; increased BDNF; reduced UPDRS motor score	Lack of placebo control; small sample size; no genetic subtyping of patients
PD	[89]	Randomised, double-blind, placebo-controlled, Phase II	Allogeneic BM-MSCs	IV	10 × 10^6^ cells/kg	Repeated injections every 18 weeks	Safe; met primary endpoint; dose–response-related	Lack of potency assays; high placebo response observed; single-centre study
ALS	[122]	Phase I/II and IIa	Autologous MSC-NTF	Intramuscular/IT	IM groups: 24 × 10^6^ cells; 36 × 10^6^ cells; 48 × 10^6^ cells IT groups: 1 × 10^6^ cells/kg; 1.5 × 10^6^ cells/kg; 2 × 10^6^ cells/kg	Phase I/II: one single administration (either IM or IT) Phase IIa: combined IM + IT administration	Safe and well-tolerated; mild and transient side effects; secondary clinically beneficial effects	Lack of placebo control; small sample size
ALS	[123]	Phase II	Autologous BM-MSCs	IT	1 × 10^6^ cells/kg	4 repeated injections (3–6 months intervals)	Safe and well-tolerated; improvements in ALSFRS-R progression;	Small sample size; lack of placebo group; high drop-out rate
ALS	[124]	Open-label, Phase I	Autologous BM-MSCs	IT	1 × 10^6^ cells per kg of body weight suspended in autologous cerebrospinal fluid per injection	2 repeated injections performed at 26-day interval	Safe and feasible; exploratory slowed decline in ALSFRS-R scores; improved FVC	Small sample size; no placebo control; lack of MSC survival and migration data

## Data Availability

Further data on the reported results are not publicly available. Additional materials related to data collection may be made available by the corresponding author upon reasonable request, who is also available for any clarifications regarding the review process or underlying the data.

## Abbreviations

AD	Alzheimer’s Disease
ALS	Amyotrophic Lateral Sclerosis
A-MSCs	Adipose Tissue-derived Mesenchymal Stem Cells
BBB	Blood–Brain Barrier
BM-MSCs	Bone Marrow-derived Mesenchymal Stem Cells
CNS	Central Nervous System
DPSCs	Dental Pulp-derived Mesenchymal Stem Cells
EVs	Extracellular Vesicles
HD	Huntington’s Disease
HTT	Huntingtin Gene
hUCB-MSCs	Human Umbilical Cord Blood-derived Mesenchymal Stem Cells
IT	Intrathecal
IV	Intravenous
mHTT	Huntingtin Mutated Protein
miRNA	MicroRNA
MSC	Mesenchymal Stem/Stromal Cell
NDs	Neurodegenerative Diseases
NFTs	Neurofibrillary Tangles
OM-MSCs	Olfactory Mucosa MSCs
PD	Parkinson’s Disease
